# Photodynamic Therapy as an Effective Treatment for Cutaneous Lymphomas

**DOI:** 10.3390/pharmaceutics15010047

**Published:** 2022-12-23

**Authors:** Stefano Caccavale, Vittorio Tancredi, Paola Vitiello, Antonello Sica, Andrea Ronchi, Renato Franco, Francesco Pastore, Giuseppe Argenziano

**Affiliations:** Dermatology Unit, Department of Mental and Physical Health and Preventive Medicine, University of Campania Luigi Vanvitelli, Via Sergio Pansini 5, 80131 Naples, Italy

**Keywords:** cutaneous T-cell lymphoma, dermatology, mycosis fungoides, photodynamic therapy, phototherapy, porphyrins, cancer, cutaneous B-cell lymphoma, pseudolymphoma

## Abstract

Topical photodynamic therapy (PDT) is a non-invasive treatment modality frequently used in dermatology to treat superficial skin cancers but also some inflammatory or infectious dermatoses. PDT appears a more and more promising therapeutic option also for cutaneous lymphomas, either of T- or B-cell origin. It is a well-tolerated treatment and has excellent cosmetic outcomes, less side effects compared to other therapies (steroids, surgery, radiotherapy, and so on), no particular contraindications, and is easily repeatable in case of relapses. However, how PDT works in the treatment of cutaneous lymphoproliferative diseases is poorly understood and the literature data are still controversial. Further randomized, controlled clinical trials involving a greater number of patients and centers with a long follow-up are necessary to assess the efficacy of PDT and establish a unique standardized treatment protocol in relation to the lymphomatous disease and the type, thickness, and location of the lesions.

## 1. Introduction

Topical photodynamic therapy (PDT) is a non-invasive treatment based on the death of cells and tissues in which the selective storage of a photosensitizer, subsequently activated by an exogenous light with a specific wavelength, has occurred. PDT was born at the beginning of the XX century, but it began to be employed in the palliative treatment of metastatic breast cancer only in 1975. Nowadays, it is predominantly used in dermatology to treat superficial skin cancers, such as actinic keratoses (AKs), superficial or nodular basal cell carcinomas (BCC), Bowen’s disease, and in situ squamous cell carcinomas (SCC). Lately, it has also been employed in the treatment of inflammatory and infectious dermatoses, such as acne, warts, and granuloma annulare [1,2,3]. 

5-aminolevulinic acid (ALA) is a small molecule formed by the enzyme ALA synthetase in the early step of the heme synthetic pathway. It is the main photosensitizer used in PDT. ALA is a zwitterion at physiological pH; being hydrophilic, it has a low ability to cross cell membranes via a passive uptake and a low selectivity and distribution in target tissues. ALA lipophilic derivatives are often used in dermatology, in particular its methyl ester methyl aminolevulinate (MAL). MAL is similar to ALA; once inside the cell, it is transformed into ALA by intracellular esterases. It was licensed for the treatment of AKs, BCC, and Bowen’s disease. Other ALA esters such as ALA hexyl ester (He-ALA) are rarely used [4]. Anyway, all these molecules are the precursors of the “true” endogenous photosensitizer protoporphyrin IX (PpIX), a fluorescent compound and the penultimate step of the heme pathway [4]. The endogenous synthesis of PpIX is controlled by the regulation of the ALA synthetase enzyme and PpIX is then converted by protoporphyrin ferrochelatase in the heme. Neoplastic cells usually have a relative deficit of ferrochelatase; thus, in tumor cells, a transient accumulation of PpIX compared to normal cells occurs [5].

PpIX in the presence of oxygen and with the appropriate wavelength of light is activated and generates active molecular species, such as free radicals and reactive oxygen species (ROS), in particular singlet oxygen, that are toxic to cells, causing direct cellular killing, modifying cellular functions, inducing cell death by necrosis or apoptosis, and vascular and local damage by immune and inflammatory mediators in target sites. 

PpIX is auto-oxidized (photobleached) by the singlet oxygen and it is consumed during the session.

PDT has also been used more and more as an off-label treatment for cutaneous lymphomas, either of T- or B-cell origin [6]. The mechanisms of the PDT efficacy in the treatment of cutaneous lymphoproliferative diseases are poorly understood and the literature data are still controversial. 

## 2. Photodynamic Therapy and Primary Cutaneous T-Cell Lymphomas

Primary cutaneous T-cell lymphomas (CTCL) are a heterogeneous group of malignant non-Hodgkin neoplasms derived from malignant T cells that traffic to the skin [4]. 

Among them, mycosis fungoides (MF) is the most frequent disease (almost 50% of all CTCL), with an annual incidence of 0.3–0.5 new cases per 100,000 inhabitants [4]. Other CTCL include Sézary syndrome, CD 30+ positive lymphomas (such as lymphomatoid papulosis and anaplastic large cell lymphoma), and the group of rare and often aggressive NK/T-cell lymphomas, such as subcutaneous panniculitis-like T-cell lymphoma and extranodal NK/T-cell lymphoma.

MF is more predominant in males and in the elderly (median age between 55 and 60 years), even though patients of all ages (children and adolescents too) may be affected. MF originates from skin-homing CD4+ T cells. Its etiology is still unknown, and the identification of genetic mutations and environmental factors requires further studies. Clinically, MF typically presents with cutaneous erythematous patches, plaques, tumors, or erythroderma. Nevertheless, atypical variants of MF exist that vary considerably in their clinical and histological features and prognosis: folliculotropic, pagetoid reticulosis, granulomatous slack skin, bullous, hypopigmented, ichthyosiform, pigmented purpuric dermatosis-like, papular, poikilodermatous, psoriasiform, pustular, and verrucoid [7,8,9,10,11]. Consequently, the diagnosis of MF is often difficult and challenging: the differential diagnosis is wide, and the histological findings may be non-specific in the early stages. Patients affected by MF may require large or multiple biopsies, as well as specialized testing and experienced pathologists to have the definite diagnosis.

Histologically, MF is characterized by the epidermotropism of lymphocytes with various degrees of atypia and intradermal lymphoid infiltrates at the beginning, which firstly invade the dermis, then the subcutaneous tissue, and lastly reach nearby organs at the advanced stages.

CTCL are usually treated with a multimodal approach. Topical corticosteroids, nitrogen mustard, carmustine, topical retinoids (bexarotene and tazarotene), local radiotherapy, surgical excision, phototherapy (UVB and PUVA), alone or in combination with systemic therapy (bexarotene, interferon, or methotrexate), and extracorporeal photochemotherapy are often used for the treatment of CTCL [4]. The selection of appropriate treatments for CTCL, in particular for MF, is fundamental and is based on the clinical stage at diagnosis and patient prognosis and comorbidities. 

**Figure 1:**
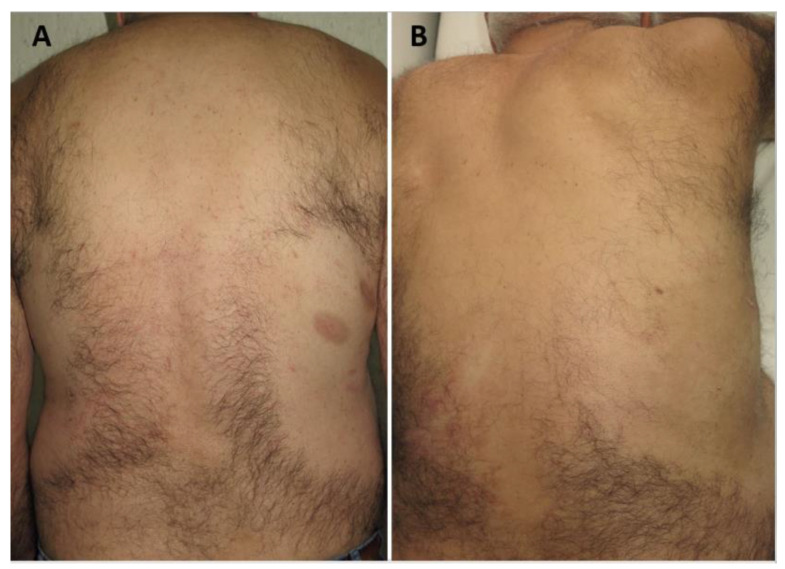
A complete response in a patient with paucilesional MF treated with 2 sessions of MAL-c-PDT. (**A**) is before the treatment, while (**B**) is after the treatment.

For patients with early stage MF, an initial treatment with local therapies is recommended, and many of these patients never need systemic therapies (Figure 1). Instead, patients with generalized forms have a high risk of progression, or even death, and often need systemic treatments. All treatments may lead to acute or chronic adverse effects and long-term toxicities that may be particularly relevant if repeated or protracted treatment schemes would be necessary (Table 1). 

Among all these therapies, clinical success was reported in the last decades using PDT to treat MF, in particular for its efficacy and its tolerability. The first case using ALA-PDT for the treatment of MF lesions was reported in 1994, since that case in 1994, many case reports of successful PDT for MF were published [24,25]. 

In a systematic review, the effectiveness of PDT on MF across twenty-four eligible studies, published between 1994 and 2017, was estimated as an overall pooled response of 69.5% [25]. Another systematic review obtained an overall complete response (CR) rate of the lesions that was 63.2% (60.9% in plaques, 72.2% in patches, and 71.4% in tumors) [26].

A recent systematic review that selectively focused on stage IA MF for the paucity of data reported a CR in 67.3%, a partial response (PR) in 13.5%, and no response (NR, defined as <50% clinical response) in 3.8% of patients. Stable disease (SD) was reported in 3.8% of cases and non-available (NA) clinical response data in 11.5% [6]. 

Moreover, PDT was reported to also be safe, non-invasive, technically simple, relatively selective, and has excellent cosmetic results, which induce a negligible general photosensitivity and no carcinogenic potential. During the illumination, patients may complain of pain, burning, and prickling, limited to the treated areas. These sensations may last a few hours after irradiation. Pain during light exposure is very variable among patients. It depends on the amount of PpIX, the light dose rate, and the total absorbed light dose. A mild or moderate erythema and edema, crusting, scaling, and a brief period of cutaneous photosensitivity are common after PDT and may last days or even some weeks. Hyper- or hypopigmentation, permanent hair loss, and scarring are rarely observed [25]. 

Obviously, adverse events depend on many variables, such as the formulation of the photosensitizer, the application time, the number and frequency of treatments, and the light dosimetry. 

There are many kinds of light sources for PDT. To obtain a maximal therapeutic effect, a light with an appropriate wavelength should be used. Visible light seems the most used in many studies of the literature. Because porphyrins have a maximum peak in their absorption spectrum at 405 nm in the blue wavelength region (Soret-band) and several weaker, longer wavelength Q-bands, the last having a peak at 635 nm in the red wavelength region, red or blue light could be used. However, because the penetration of light into skin is related to wavelength, red light has better tissue penetration compared to the blue light. The latter is most suitable for thin lesions. In fact, below approximately 600 nm, the hemoglobin and melanin absorption limit the light that reaches the dermal capillaries. Therefore, the most used artificial light source for MF in the literature is usually red light at around 630 nm, which guarantees an optimal tissue penetration to the dermis, also covering the spectrum of the PpIX absorption band (conventional PDT, c-PDT). Probably, long wavelengths achieve better penetration than short wavelengths and are consequently more effective for thicker MF lesions [6]. However, many other sources of light could be used in clinical practice, such as lights of different wavelengths, lasers, incoherent light sources, and even daylight (daylight PDT or DL-PDT). In addition, the best light exposure dose or fluence (J/cm^2^) and the light intensity or irradiance (mW/cm^2^), which are important parameters to be selected during every session of PDT, remain to be determined for MF. 

With regard to the selection of the photosensitizer, MAL is reported to have an increased lipophilicity, and therefore also a deeper skin penetration through membranes, and a higher PpIX synthesis compared to ALA. As a result, MAL-PDT is considered more selective toward neoplastic cells, and it allows a shorter incubation time compared to ALA-PDT [25]. Anyway, there is an increasing need to develop and use new and more specific photosensitizers for this disease. 

The total number of PDT sessions and the time interval between two treatment sessions in the literature is very variable for MF (1 to 14 PDT treatments), because established treatment protocols of PDT for MF or CTCL have not yet been optimized [25]. A mean number of 9.5 was reported in a recent systematic review (range 1–46) [6]. Certainly, PDT needs more sessions to be successful in treating MF. The frequency of sessions is about every 1 to 8 weeks in the literature [25]. Certainly, it is related to the clinical response and the variability in the clinical presentation [25]. 

Even the lesion location may alter the clinical response to PDT. In fact, the efficacy of PDT is reduced when the photosensitizer occlusion is more easily interrupted by patient movements (e.g., the inguinal and gluteal regions, hands, and feet) [6]. Moreover, skin thickness is related to PDT efficacy and is probably different among ethnicities [6]. 

**Figure 2:**
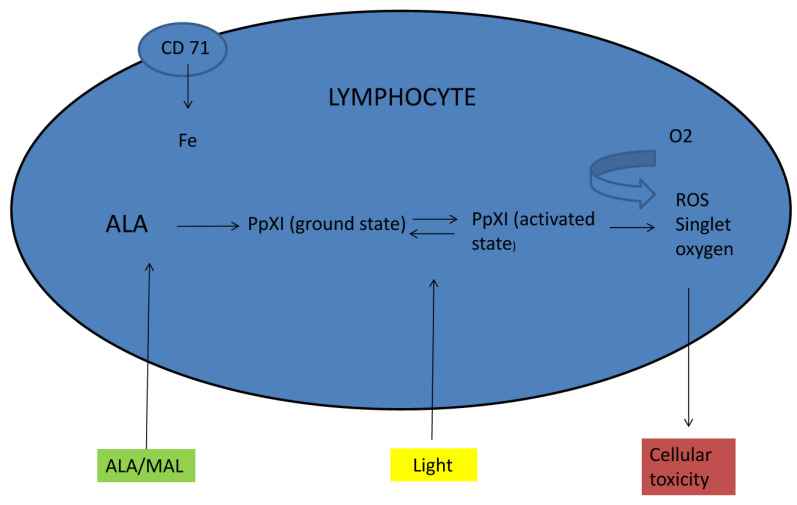
Schematic image of PDT effects in malignant lymphocytes of MF.

How PDT works in treating MF is poorly understood because the contribution to neoplastic cell death and the role of inflammatory cells in the response has not been elucidated. Proliferating tumor cells are certainly more susceptible to photosensitizers. PpIX production is probably increased in highly proliferating tissues, such as T cutaneous lymphomas, by changes in the cellular enzyme activity, leading to a strong reduction in the malignant cells number and the suppression of proliferation. In addition, the high expression of the transferrin receptor (CD71) on the surface of malignant lymphocytes might induce a higher turnover of iron, leading to increased PpIX production [26,27] (Figure 2). Moreover, PDT seems to have a direct potential for the induction of toxicity in T cells. In 2001, Gad et al. demonstrated an increase in caspase-3-like activities and an increase in the DNA fragmentation in malignant T cells following PDT [26,28]. 

PDT is mainly used for the treatment of early stage (patches or plaques), unilesional, or paucilesional MF lesions, especially in the case of relapses or resistant lesions or in sensitive areas such as the face and neck [4]. It can be used alone, as a primary therapy, or as a component of combination regimens. Instead, PDT use is still controversial for tumor-stage and erythrodermic MF, as well as for Sezary syndrome [4]. A limitation to the use of both ALA-PDT and MAL-PDT in MF is the poor tissutal penetration of the photosensitizers that is insufficient in the tumor stage, thus inducing an insufficient cellular necrosis. Moreover, patients need to wait for a long incubation period (about 3 h for MAL and even more for ALA) between the photosensitizer application and light exposure so that PpIX is produced and accumulated within the neoplastic cells (Figure 2). 

## 3. Photodynamic Therapy and Primary Cutaneous B-Cell Lymphomas

Primary cutaneous B-cell lymphomas (CBCL) are a heterogeneous group of extranodal B-cell non-Hodgkin lymphomas, which primarily involve the skin without evidence of extracutaneous disease at the onset [29]. Among them, different subtypes have been identified. Despite being rare diseases, their incidence has increased in the last years and currently is about four cases for one million people, with a middle age of onset >50 years [29].

The three major subtypes are (WHO 2017) primary cutaneous marginal zone lymphoma (PCMZL), primary cutaneous follicle center lymphoma (PCFCL), and diffuse large B-cell lymphoma, leg type (PCDLBCL, LT) [30].

PCFCL is the most common type [30]. It is slightly more common in males than in females and its etiology is unknown [30,31]. It is a low-grade and non-aggressive lymphoma [29,30,31]. Clinical manifestations of PCFCL consist of a unique firm papule or nodule; sometimes there are multiple lesions but in a cluster [31]. Most of the time, lesions interest the upper part of the body, such as the head–neck region [31]. Histologic features are a dermal infiltration, which resembles follicular structures, a monoclonal centroblast proliferation, and an abundant T-cell-reactive contingent. Neoplastic cells are usually CD20+, bcl6+, and bcl2− [31].

PCMZL is another subtype of non-aggressive CBCL [29]. Its prevalence is higher in males than females [29,30]. The etiology of PCMZL has been the object of discussion, and in some patients, a possible role of Borrelia burgdorferi infection has been supposed [32]. Clinical manifestations consist of pink or reddish papules mainly affecting the trunk and upper limbs [30,33]. PCMZL is an indolent lymphoma, and its prognosis is usually benign [29,33]. The histology shows a nodular or diffuse proliferation of B-derived cells, which are often CD20+, bcl2+, bcl6-, and CD10-, with epidermal sparing [33]. 

For both PCFCL and PCMZL, an extracutaneous involvement is very rare, and the overall prognosis is excellent (5-year survival rate > 95%) [34]. The therapeutic approaches are similar for these two variants of CBCL and depend on the number of lesions, the age and compliance of each patient, the presence of a systemic lymphoma or negative prognostic factors, comorbidities, and so on. Usually, surgical excision, when the number of lesions is limited, and radiotherapy (RT) represent the gold standard, with a high rate of response [31,32,34]. The RT feasibility is linked to the number of lesions and the possibility to include all of them in a single area of irradiation. Despite effective treatments, about 25% of cases have relapses [35]. Many therapies have been proposed, including imiquimod, cryotherapy, topical and intralesional steroids, the antiCD20 monoclonal antibody, and chemotherapy [36]. In the case of a Borrelia burgdorferi infection, the therapy includes antibiotic intake [33,37]. 

Rarely, off-label PDT has been used for low-grade CBCL treatment. Within all the literature currently available on Pubmed, we found only a pilot study published in 2006 [38], in which three patients with an indolent CBCL were treated with PDT. One of these patients was affected by a PCFCL and the others by PCMZL. The photosensitizing agents used for these patients were ALA 20% (water in oil emulsion) and MAL [38]. In all the cases, a 4-h occlusion time was performed. A 630 nm wavelength light, emitted by a diode lamp, was used at a fluence of 37 J/cm^2^ for the session. All the treated patients had complete remission, defined as a clinical and histologic complete absence of CBCL, after a maximum of two PDT sessions at a 1-week interval. No patient experienced particular side effects, and the pain was easily managed after the PDT sessions [38].

PCDLBCL, LT is the most frequent subtype of diffuse large CBCL [29]. It is more common in elderly women [30]. The clinical presentation consists of multiple nodules or plaques, often ulcerated, on the legs, sometimes bilateral but usually not symmetric [39]. About 10% of patients have an extra leg skin disease, and an extracutaneous involvement is frequent [39]. The histology pattern is characterized by a massive dermal large cell proliferation that may involve the hypoderm too. The neoplastic proliferation is CD20+, bcl-2+, CD79+, MUM1+, and CD10-. Reactive T-cell proliferation is often absent [39]. The course of this lymphoma is similar to the classic diffuse large B-cell lymphoma; thus, its prognosis is usually severe, with a 5-year survival rate < 60% [29,34,39]. Being an aggressive type of lymphoma, the therapy consists of systemic chemotherapy, and the R-CHOP (rituximab, cyclophosphamide, doxorubicin, vincristine, and prednisone) scheme is one of the most used [34,39]. Radiotherapy (RT) can be associated, while no experience in the literature is reported with PDT [34,39].

## 4. Cutaneous Pseudolymphomas

The term cutaneous pseudolymphoma (CPL) refers to some cutaneous benign entities characterized by a diffuse lymphocytic proliferation that histologically and often clinically resembles a real cutaneous lymphoma [40]. CPL is considered a sort of benign inflammatory response to some triggers (insect bites or stings, drugs, tattoos, vaccination, and so on). Usually, CPL are divided into B-cell CPL (some of them are also known under the name of lymphocytoma cutis, or lymphadenosis benigna cutis, or cutaneous lymphoid hyperplasia) and T-cell CPL, if they resemble, respectively, a B or T lymphoma [40,41].

The typical clinical manifestation is a smooth papule, nodule, or plaque on the head or neck. However, CPL may also present as multiple or widespread lesions. Their color is often red, violaceous, or the same of the skin.

The treatment is not standardized and depends on the specific form of CPL: the most common therapies include topical and systemic steroids, cryotherapy, local radiotherapy, and surgical excision [42].

PDT has never been evaluated in a clinical trial with a controlled group for CPL treatment; anyway, few case reports are available in the literature.

Takeda et al., in 2005, treated two patients affected by lymphadenosis benigna cutis of the face with PDT. The photosensitizer used by the authors was ALA 20% (an oil–water emulsion) applied with an occlusion medication protected by the light for 4–6 h and then irradiated with visible light (peak 630 nm) for 15–20 min at a single session dose of 120 J/cm^2^. The PDT was repeated every two weeks for five sessions [43].

Mikasa et al., in 2005, published a case report on the use of 20%-ALA-PDT in a 51-year-old female patient with two B-cell CPLs localized on her right cheek and nose. After 4–6 h of an occlusion medication, the lesions were irradiated with a pulsed 630 nm artificial visible light, at a dose session of 100 J/cm^2^, by using an excimer dye laser. The cheek lesion was irradiated 3 times, while the nasal one was 5 times. The clinical results were remarkable, but the patient refused a control punch biopsy [44].

In 2020, our group published a case of CPL in a 50-year-old patient treated with MAL-PDT [45]. MAL cream was applied and after 3 h of occlusion without light exposition, an artificial red light of 630 nm (Aktilite) was administrated at a dose of 100 J/cm^2^ (about 20 min). The treatment was repeated every 3 weeks for a total of three sessions. A full clinical response was obtained, and our patient was disease free after 12 months of follow-up [45].

Not only ALA or MAL have been used as photosensitizers. In 2020, Olisova et al. used chlorin e6 for the treatment of five cases of B-cell CPL. After 30 min from the application, the patients were irradiated with a diode lamp at a wavelength of 665 ± 10 nm for 40 min, weekly, for 4 ± 2 sessions in total. The majority of the patients obtained a complete remission (confirmed after 8 months of clinical follow-up). Only one patient had a partial response [46]. Some patients complained of pain (considered mild in its peak), erythema, and transient hyperpigmentation after the PDT.

## 5. Conclusions

PDT has given promising results in many reports published in the literature. PDT confirms itself as a safe and effective treatment for cutaneous lymphomas and pseudolymphomas, which has fewer side effects compared to other therapies (steroids, surgery, radiotherapy, and so on), no particular contraindications, and is easily repeatable in case of relapses. It is a well-tolerated option and has excellent cosmetic outcomes. The main side effects are acute and not severe. A burning sensation during the session is frequent; some patients may complain about severe pain which may require treatment interruption. Contraindications are represented by hypersensitivity to the photosensitizer used, pregnancy or lactation, porphyria, lupus erythematosus, and other photosensitivity dermatoses. Moreover, PDT use should not be preferred in the case of multiple and widespread lesions, especially when it could be difficult or impossible to include all of them in a few illumination fields. PDT should be considered only by specialists that are experts in the treatment and management of mycosis fungoides and other cutaneous lymphomas because identifying the stage of the disease and the type of lesions is of crucial importance to decide which patients can benefit from PDT.

However, further randomized, controlled clinical trials involving a greater number of patients and centers with a long follow-up are necessary to assess the efficacy of PDT and establish a unique standardized treatment protocol in relation to the lymphomatous disease, the lesion type, thickness, and location. Principal aspects to define concern the type of photosensitizer, the duration of occlusion, the wavelength and the type of light dispenser, the energy required, the number of sessions needed, and how to properly prepare the lesions for the treatment. New frontiers and horizons of PDT are represented by innovative photosensitizers beyond ALA and its esters, different ways to deliver the photosensitizers by injection or nanosized carriers, and the employment of prepping techniques such as microneedling. In addition, further studies should be conducted to assess the response to PDT by means of non-invasive diagnostic techniques, such as confocal microscopy, line-field confocal optical coherence tomography (LC-OCT), and high-frequency ultrasonography.

## Figures and Tables

**Table 1:** Recent literature on the use of PDT on primary cutaneous T-cell lymphomas.

Investigator	Type of MF	Number of Patients/Lesions	Lesion Type	Previous Treatments	Photosensitizer/Occlusion Time (h)	Light (nm)/Light Doses (J/cm^2^)	Number of PDT Sessions	Complete Response (%)	Follow-Up (mo)
Dairi et al. [12]	MF, FMF	4/4	Plaque	Clobetasol	16% MAL/3	630/37	4–12 AFL-PDT	4/4 (100%)	6–18
Debu et al. [13]	FMF	3/8	Plaque/1Patch	NM	16.8% MAL/3	630/37	1–7	7/8 (88%)	12–28
Pileri et al. [14]	MF	4/4	Patch	PUVA and acitretin, topical and systemic steroids, nbUVB	16% MAL/3	630/37	4–9	2/4 (50%) and 2/4 PR (50%)	6–120
Jung et al. [15]	Localized pagetoid reticulosis	1/1	Plaque	Eight treatment sessions with a 308 nm excimer laser	16% MAL/1.5	630/37	8 AFL-PDT	1/1 (100)	NM
Jang et al. [16]	MF	1/1	Patch	Topical steroids	MAL cream/4	570–670/37	2	1/1 (100%)	NM
Calzavara-Pinton et al. [17]	MF	19/19	Plaque	NM	16% MAL/3–4	635 ± 18/37	1–7	5/19 (26%); 2 patients relapsed at follow-up	10.0±10.5
Quereux et al. [18]	MF	12/29	Patch, Plaque	HN2, BCNU, nbUVB, Rx, imiquimod, PUVA, systemic bexarotene, interferon	20% MAL/3	630/37	2–6	6/12 (50%) and 3/12 PR (25%)	6–35
Han et al. [19]	MF	3/3	Plaque	PUVA, interferon	20% ALA solution/4	635/60 nw/cm^2^	2–3	2/3 (66.7%) and 1/3 PR (33.3%)	8–17
Kim et al. [20]	MF	10/10	Plaque, Patch	UVA1, acitretin, PUVA, topical steroids	16.8% MAL/3	630/37.5	2–6	5/10 (50%) and 2/10 PR (20%)	8–31
Kaufmann et al. [21]	MF	1/1	Patch	Topical steroids and topical immunomodulators	20% ALA/	Cooled LED-based PDT device	8	1/1 (100%)	48
Hasson et al. [22]	MF	1/1	Tumor	PUVA, bexarotene, TSEBI	16% MAL/3	Incoherent light, 570–670 nm/37 J/cm^2^/70 Mw/cm^2^	3	1/1 (100%)	60
Juan-Carpena et al. [23]	MF	1/1	Plaque	Topical cor-ticosteroids, oral acitretin, PUVA, oral methotrexate and interferon, imiquimod, HN2	16% MAL/NM	630/37 (c-PDT on palms) and DL-PDT (soles)	6 c-PDT (palms) and 8 DL-PDT (soles)	4/4 (100%)	12

CR: complete response; PR: partial response; NM: non-mentioned; MF: mycosis fungoides; FMF: folliculotropic mycosis fungoides; MAL: methyl-aminolevulinic acid; ALA: aminolevulinic acid; nbUVB: narrowband ultraviolet B; PUVA: psoralen ultraviolet A; UVA1: ultraviolet A1; AFL: ablative fractional CO2 laser; HN2: mechlorethamine; BCNU: carmustine; Rx: radiotherapy; TSEBI: total-skin electron beam irradiation.
